# Curved surface effect and manipulation of electronic states in nanosilicon

**DOI:** 10.1038/s41598-017-18377-9

**Published:** 2017-12-21

**Authors:** Zhong-Mei Huang, Wei-Qi Huang, Xue-Ke Wu, Shi-Rong Liu, Cao-Jian Qin

**Affiliations:** 1 College of materials and metallurgy, Institute of Nanophotonic Physics, Guizhou University, Guiyang, 550025 China; 2 State key laboratory of Surface Physics, Key Laboratory of Micro and Nano Photonic Structures (Ministry of Education) and Department of Physics, Fudan University, Shanghai, 200433 China; 3 State Key Laboratory of Environmental Geochemistry Institute of Geochemistry, Chinese Academy of Science Institute of Geochemistry, Guiyang, 550003 China

## Abstract

It is interesting in low-dimensional nanostructures of silicon that the two quantum effects play different roles in nanosilicon emission, in which the quantum confinement (QC) effect opens band gap and makes emission shift into shorter wavelengths (blue-shift) as the size of the nanocrystals is reduced; however the breaking symmetry originating from impurities on nanosilicon produces the localized electronic states in band gap and makes emission shift into longer wavelengths (red-shift). The results of experiment and calculation demonstrated that the energy levels of nanosilicon can be manipulated through these quantum effects, where the curved surface (CS) effect of impurity atoms bonding on nanosilicon is important in breaking symmetry of nanosilicon system. Here, the CS effect plays an important role on impuritied nanosilicon in smaller scale with larger surface curvature, in which a few characteristic parameters have been found to describe the breaking symmetry of nanosilicon system, such as bonding angle and projecting length of bonds on curved surface. More interesting, the coupling ways between the QC effect and the CS effect determinate the levels position of localized states in band gap and manipulate emission wavelength, where a few new phenomena were explored.

## Introduction

Despite bulk silicon has an indirect band gap, rendering the emission in the material relatively inefficient because of accompany by phonons for conservation of momentum, the significant progress has been made in the development of silicon photonics^1–12^. The efficient way to manipulate the energy structure of silicon is through quantum confinement (QC) effect in nanostructures^13–16^, and photoluminescence (PL) emission excited on nanosilicon occurs a blue shift and an increase in intensity as the size of the nanocrystals is reduced, which reflect the opening up of the band gap^17,18^ and enhancement in the radiative recombination rate of electron–hole pairs as momentum conservation is gradually relaxed due to the Heisenberg uncertainty principle related to Δp ~ h/Δx^19^. The notable applications in photonics^20^, photovoltaics^21,22^ and highly efficient solar cells^23,24^ have been proposed.

However, the wavelength of PL emission stands at some fixed positions and even shifts into longer region for smaller nanosilicon prepared in various impurities environment^25,26^. De Boer *et al*. reported experimental evidence for a short-lived visible band in the PL spectrum of silicon nanocrystals in which the emission intensity increases and the emission shifts into longer wavelengths (red-shift) with smaller nanocrystal sizes^27^. In some experiments, when embedded in an oxide matrix, the resulting non-planar nanocrystal/oxide interface provides an exemplar system to evidence the competition between quantum confinement and surface states effects in silicon nanostructures^28^. Numerous models have been proposed to explain the change of the PL spectra. Wolkin *et al*. indicated that the disappearing of the blue-shifting is related to the trapping of an electron by Si = O bond which produces localized states in the band gap of nanoslicon smaller than 3 nm^29^.

The central questions are why some bonds on the surface of smaller nanosilicon can produce localized states in gap to break the QC effect and what relation between the localized levels and the shape of nanosilicon it is. Hadjisavvas *et al*. found that the shape of larger nanocrystals is often observed to be faceted, while the shape of smaller ones is always spherical^30^, from which we can reveal the reason why the curved surface (CS) effect^31,32^ plays a main role on smaller nanosilicon. In the CS effect, the impurity atoms bonding on smaller nanosilicon could obviously break the symmetry of nanosilicon system, which couples with the QC effect to determinate the energy levels of localized electronic states in band gap and manipulate emission wavelength. The energy E_L_ of localized levels on nanosilicon with coupling of the QC effect and the CS effect can be described by the formula: E_L_ = C/r^m^ + βB^1/(d+1)^ /R, where the first term relates to the QC effect and the second term relates to the CS effect^31^, in which R is the curvature radius of surface and B is the bonding cover factor on surface whose index d is cover dimension, such as d = 0 for Si = O bond, d = 1 for Si-O-Si bond, and d = 2 for Si-N bond. They relate to point, line, and face forms of bonding cover, respectively. In the second term,βis an experimental factor in the CS effect. In previous work, the CS effect is often submerged in the size effect, which makes some confusion when the QC effect fails for smaller nanosilicon.

In the article, the investigation results demonstrated that the energy levels of nanosilicon can be manipulated through the quantum effects, where in the impurity atoms bonding on nanosilicon is important in breaking symmetry of nanosilicon system. Here, the CS effect plays an important role on impuritied nanosilicon with smaller scale and larger surface curvature. In simulating calculation, a few characteristic parameters have been found to describe the breaking symmetry of nanosilicon system, such as bonding angle and projecting length of bonds on curved surface. It is interesting that the coupling ways between the QC effect and the CS effect determinate the position of localized electronic states in band gap and manipulate emission wavelength.

In the Wolkin *et al*. calculation, the localized states could not be found in band gap as Si-O-Si bridges bonding on the nanosilicon^29^. Our calculation results show that the localized states can be produced in band gap by Si-O-Si bridge bond on the curved surface of smaller nanosilicon (<2 nm), but no any localized state occurs in gap for Si-O-Si bridge bonding on the facet of larger nanosilicon (>2.5 nm)^31^. Therefore, besides size, shape of nanosilicon is more essential for producing localized state in band gap. In the article, the simulating calculation on nanosilicon with spherical shape in diameter scale from 0.6 ~ 1.8 nm exhibits the detail change of the localized states in band gap, in which the a few characteristic parameters have been selected to describe the breaking symmetry of nanosilicon system, such as bonding angle and projecting length of bonds on curved surface. It is interesting that these characteristic parameters can obviously change the levels position of the localized states in band gap independent of nanosilicon size, where some ruler may be defined to characterize the breaking symmetry of nanosilicon system.

**Figure 1:**
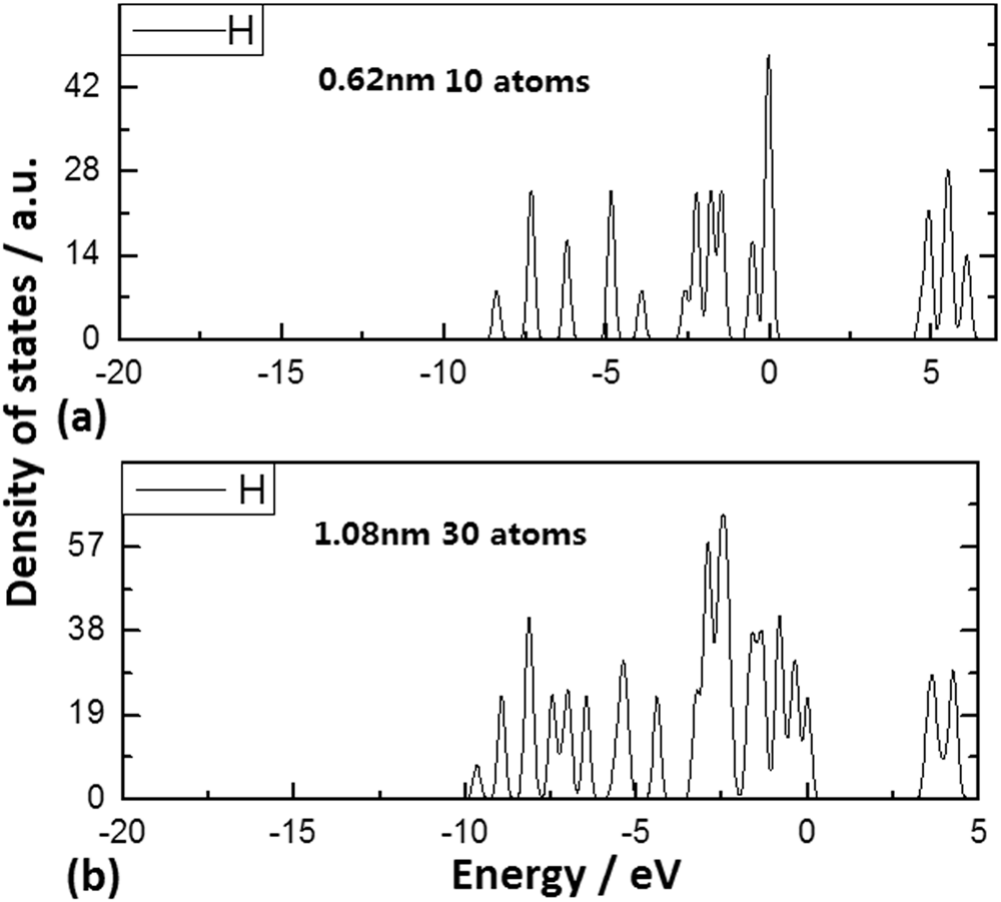
DOS of Si QDs structures with a passivation of Si-H bonds in simulating calculation. (**a**) DOS distribution of Si QD with Si-H bonds passivation, involving 10 Si atoms with diameter of 0.62 nm. (**b**) DOS distribution of Si QD with Si-H bonds passivation, involving 33 Si atoms with diameter of 1.08 nm.

In order to reveal the QC effect and the CS effect, the detail simulating calculation (see method) was carried out with various diameters on smaller Si QDs doped with oxygen or nitrogen, where the related parameters such as bonding angle and projection length of bonds on surface have been chosen to show the relations to the levels change of the localized states. In Fig. 1, the Si QDs structure with a passivation of Si-H bonds can almost be kept in the symmetry of the nanosilicon system, but on the too small QD structure (diameter < 0.6 nm) passivated with Si-H bonds the localized states begin to enter the band gap as shown in Fig. 1(a) where the Si QD involves 10 atoms with diameter of 0.62 nm. And Fig. 1(b) exhibits the DOS of the Si QD involving 33 atoms with diameter of 1.08 nm, in which there is almost no localized state in the band gap. Here, the QC effect plays a main role in the symmetric nanosilicon system.

**Figure 2:**
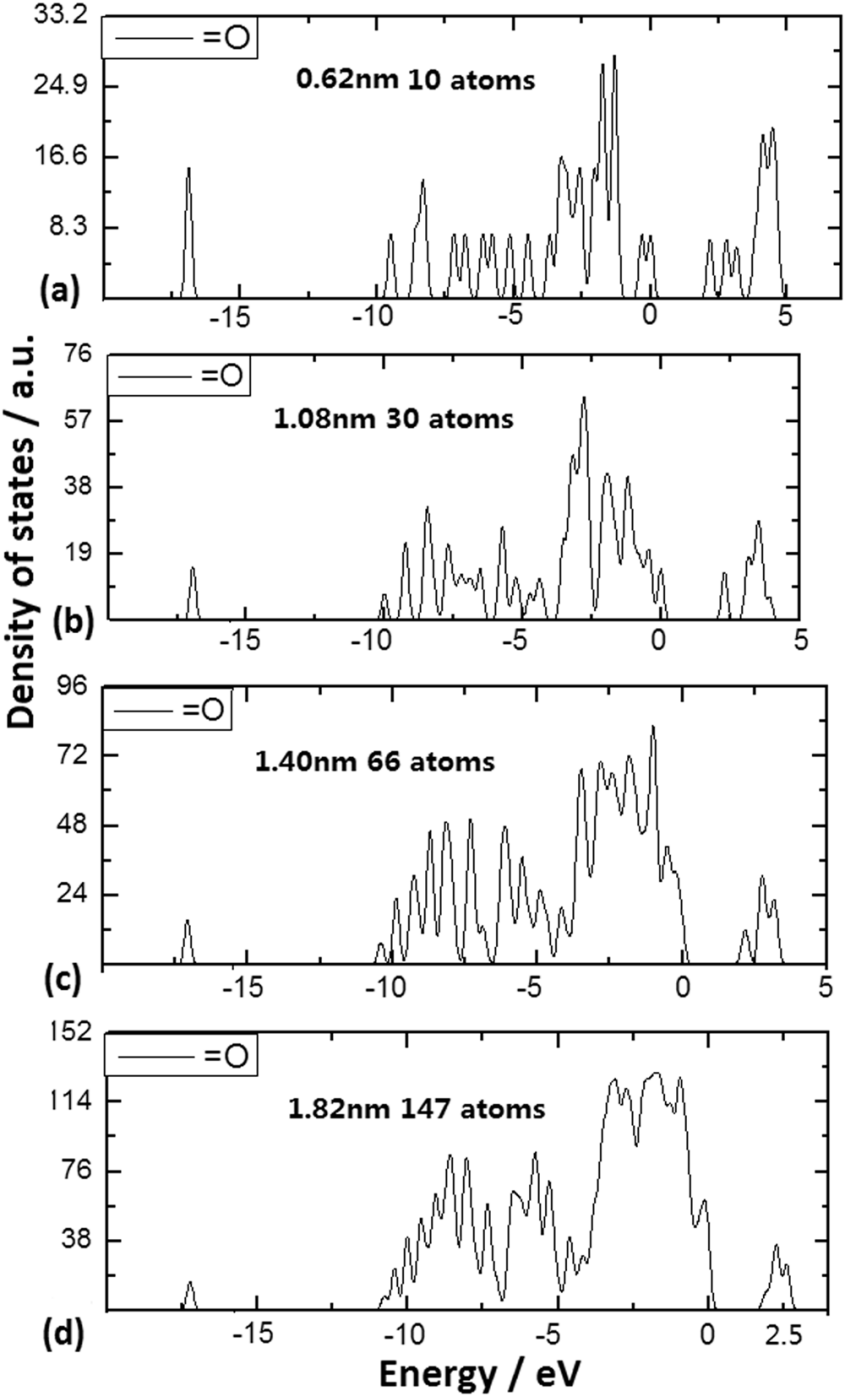
DOS of Si QDs structures with Si = O bonds on curved surface, obviously breaking symmetry of nanosilicon system, in which the localized states occur in band gap. (**a**) DOS of Si QDs involving 10 Si atoms with Si = O bonds on surface. (**b**) DOS of Si QDs involving 33 Si atoms with Si = O bonds on surface. (**c**) DOS of Si QDs involving 66 Si atoms with Si = O bonds on surface. (**d**) DOS of Si QDs involving 147 Si atoms with Si = O bonds on surface.

The Si = O bonds on QDs surface obviously break the symmetry of the nanosilicon system, in the CS effect, the change of the localized states on impuritied QDs involving 10, 33, 66 and 147 Si atoms is shown respectively in Fig. 2 (a,b,c and d). It should be noted that the localized states begin to disappear in band gap on the QD involving 147 Si atoms as shown in Fig. 2(d). It is interesting to make a comparison for localized states change with increasing diameter of impuritied QDs, in which the competition between the QC effect and CS effect occurs in nanosilicon bonding with Si = O bonds on surface. Here, in the CS effect formula, the parameter d is 0 and the formula is described as following: E_L_ = C/r^m^ + βB /R, where m is about 1.7 and β are experimental parameters, the bonding cover factor B should be related to projection of bonds on curved surface, in which there is the strongest CS effect owing to the biggest term βB/R related to the abundant localized states in the band gap as shown in Fig. 2(a). Here, we can see the extant coupling between the QC effect and the CS effect, in which the QC effect opens the band gap to provide a wider space in the gap for the localized states in the CS effect.

**Figure 3:**
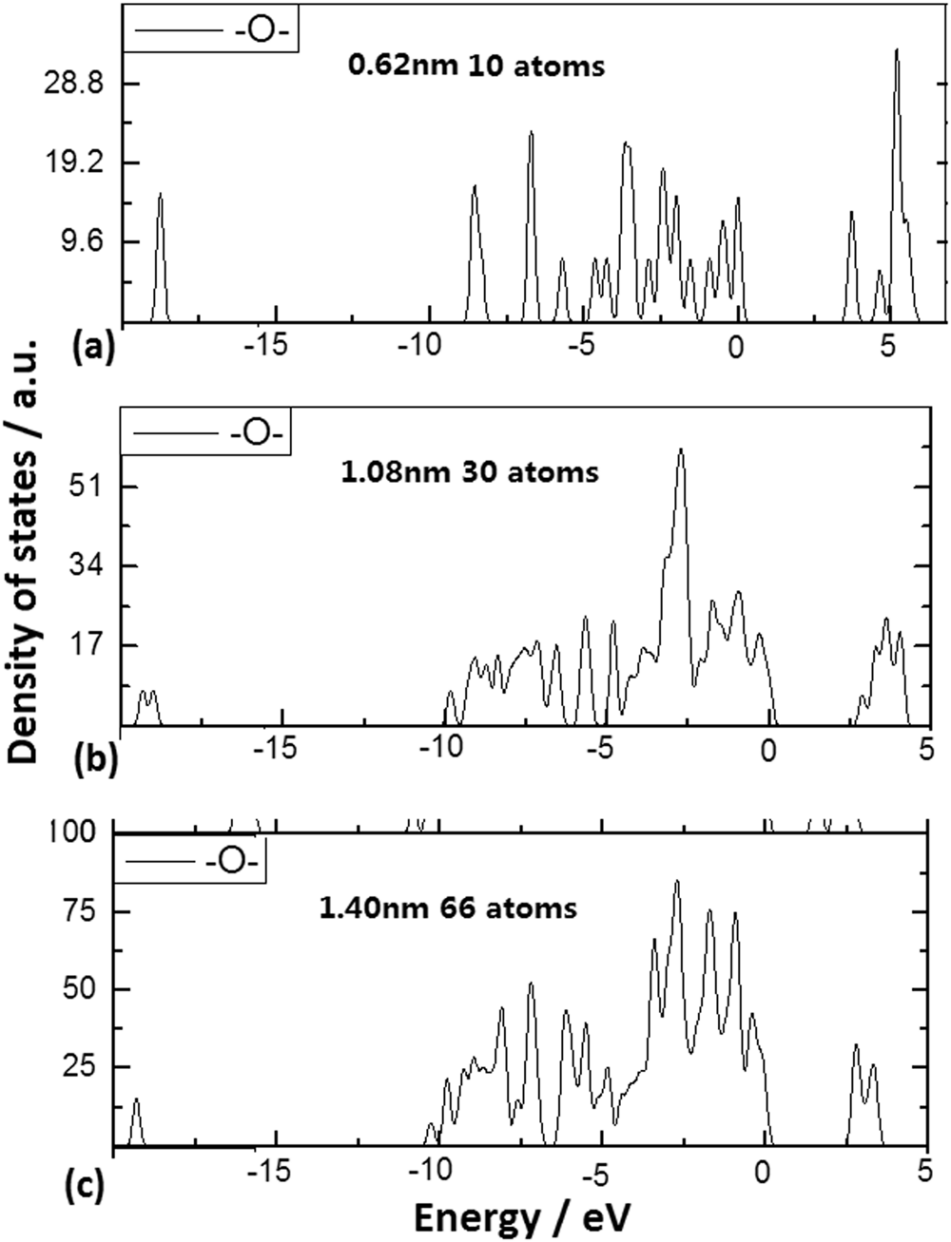
DOS of Si QDs structures with Si-O-Si bonds on curved surface. (**a**) DOS of Si QDs involving 10 Si atoms with Si-O-Si bonds on surface, in which the localized states occur in band gap. (**b**) DOS of Si QDs involving 33 Si atoms with Si-O-Si bonds on surface, in which the localized states occur in band gap. (**c**) DOS of Si QDs involving 66 Si atoms with Si-O-Si bonds on surface, where the localized states begin to disappear in band gap as QD diameter is near 2 nm.

In the same way, the Si-O-Si bonds on QDs surface can obviously produce the change of the localized states in band gap, as shown in Fig. 3(a,b and c) relating to the QDs with 10, 33 and 66 Si atoms respectively. The abundant localized states occur in the band gap on impuritied QD of 10 atoms with bigger surface curvature, while the localized states begin to disappear in band gap on impuritied QD of 66 atoms with smaller surface curvature. Here, in the CS effect formula, parameter **d** equals to 1 and the formula is described as following: E_L_ = C/r^m^ + βB^1/2^ /R, where the bonding cover factor B should be related to bonding angle and projection length of bonds on curved surface.

**Figure 4:**
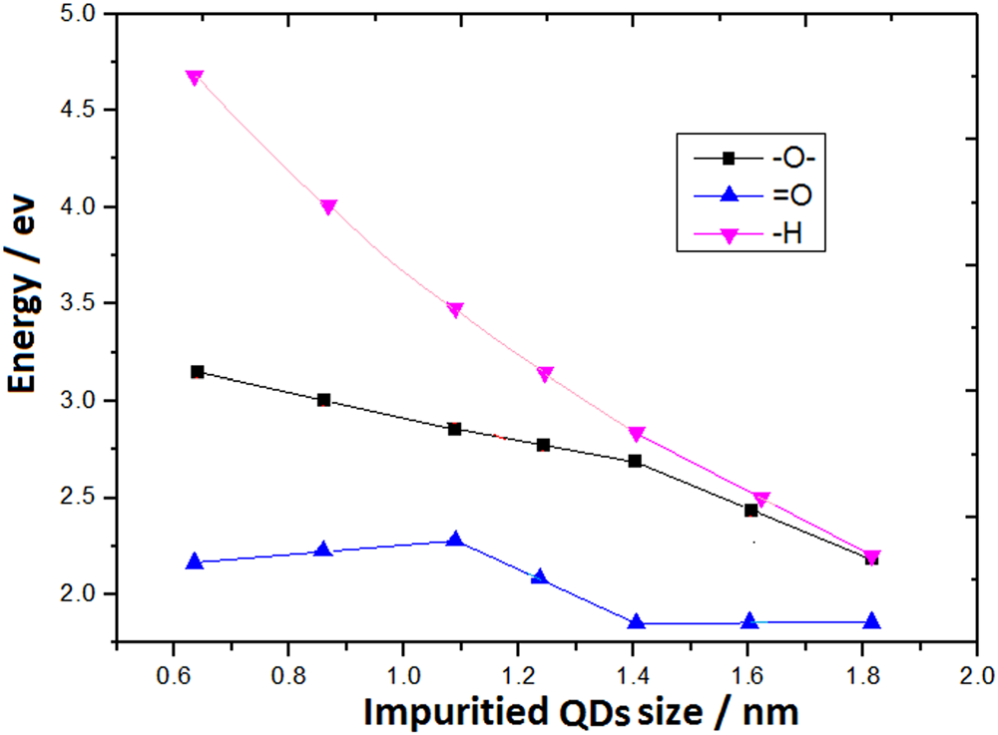
Levels evolution of formation energies for each of the investigated Si QDs with Si-O-Si bonds, Si = O bonds and Si-H bonds passivation on surface, where the Si = O and Si-O-Si bonds obviously break the QC effect.

The calculated formation energies for each of the investigated Si QDs impuritied, with different types of surface bonds, are shown in Fig. 4. It is noticeable that the Si = O bonds have the biggest CS effect on the formation energy and the Si-O-Si bonds on surface can also significantly decrease the energy levels comparing with the Si-H bonds doing. Here, the CS effect on smaller QDs with Si = O and Si-O-Si bonds obviously breaks the QC effect. The lower levels show that impurities with Si = O and Si-O-Si bonds on QDs are the better stable nanosilicon.

### Characteristic parameters in the CS effect

**Figure 5:**
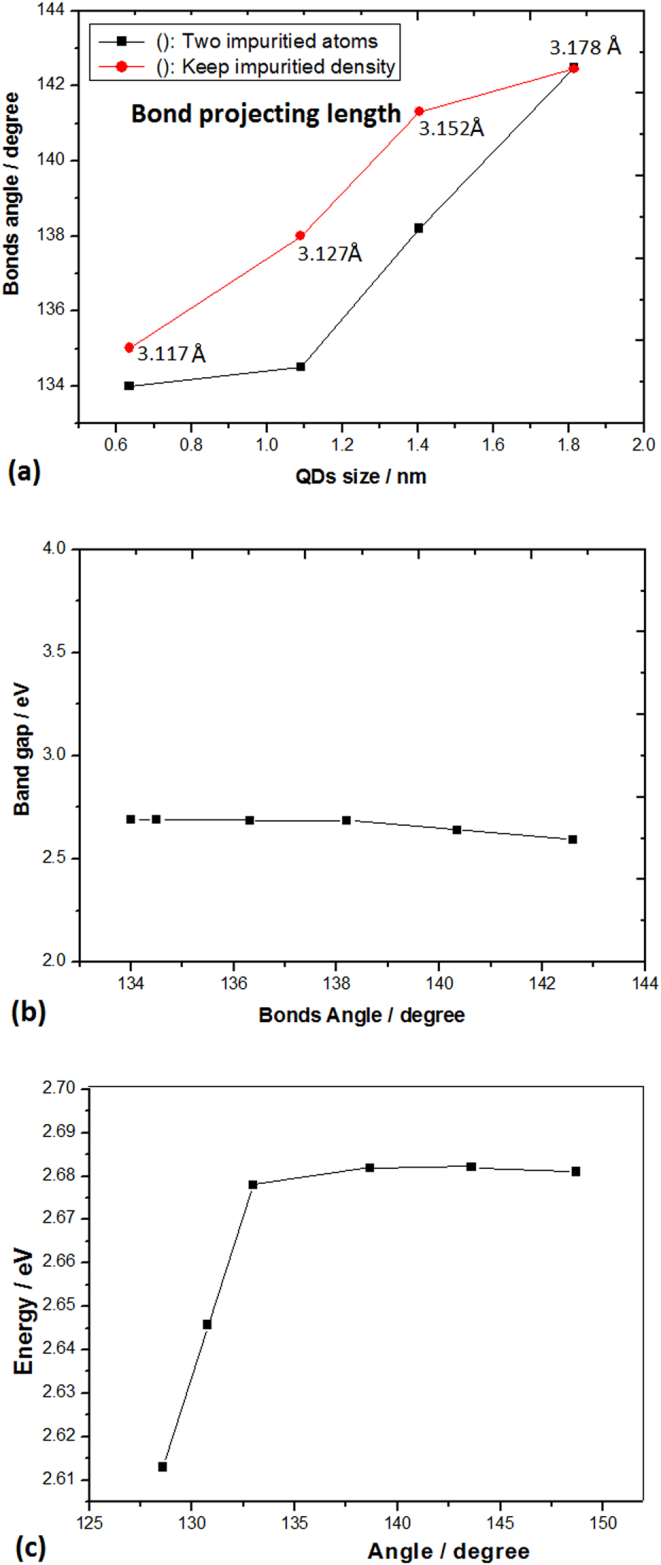
(**a**) Change of bonding angle and bonds projection length on surface with decrease of QDs size and increase of surface curvature, where the red curve relates to keeping the impurities density on surface and the black curve relates to just two Si-O-Si bonds on various surface. (**b**) Levels change of localized states with the increasing bonding angle on the QD surface with standing diameter of 1.4 nm, which belongs to the pure CS effect. (**c**) Energy levels descending of localized states with decrease of bonding angle and increase of surface curvature extensively, where the pure CS effect is obviously exhibited.

More interesting, the bonding angle and the projection length of Si-O-Si bonds on surface have been found for change of the levels position with decrease of the QDs size and increase of surface curvature in the calculation. It is discovered that the bonding angle decrease of Si-O-Si bonds with increase of surface curvature on QDs plays a main role in the CS effect, as shown in Fig. 5. Figure 5(a) shows the levels position decline with decrease of the bonds projection length on surface and the QDs size, in which the red curve relates to the evolution as keeping the impurities density on surface and the black curve relates to the evolution as just two Si-O-Si bonds on various surface. And Fig. 5(b) exhibits the levels change of localized states with increase of surface curvature and decrease of bonding angle of Si-O-Si bonds on QD surface at standing diameter of 1.4 nm (independent of size), which belongs to the pure CS effect. It should be noted that the levels position of localized states deeply descends with decreasing bonding angle extensively to 128.5 degree, as shown in Fig. 5(c), where the pure CS effect is obviously exhibited.

### Competition between the QC effect and the CS effect

**Figure 6:**
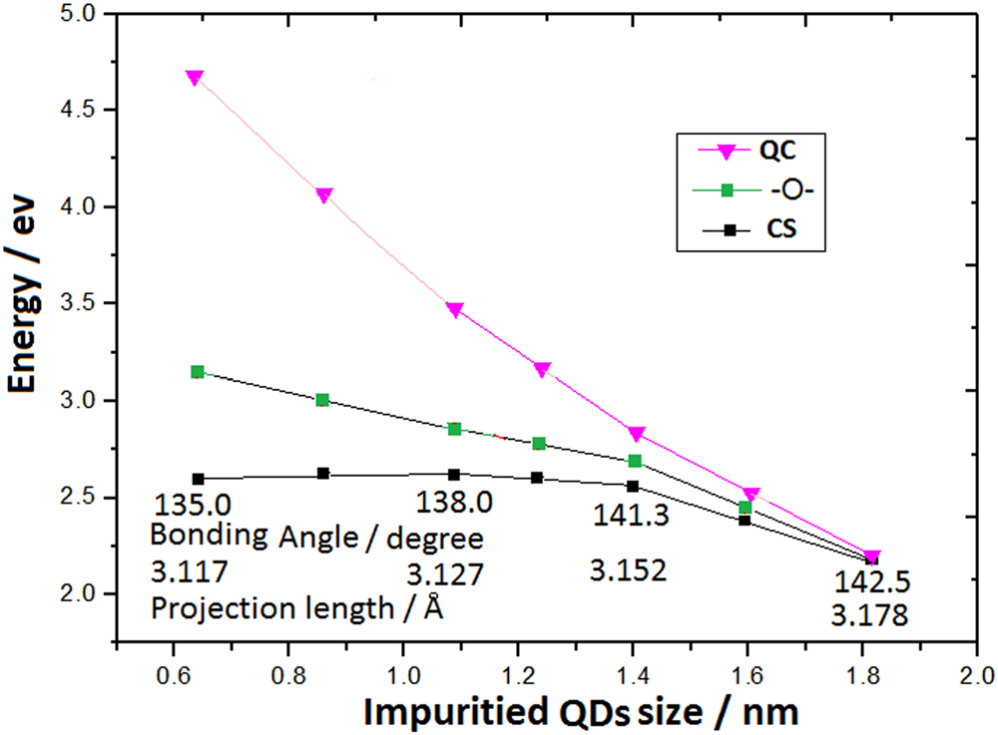
Competition between the QC effect and the CS effect on Si QDs doped with Si-O-Si bonds on curved surface, where the red curve and the black curve respectively describe the QC effect and the CS effect evolution, and the green curve is the last evolution after coupling between the QC effect and the CS effect, in which the characteristic parameters of the CS effect are marked along with the black curve.

Figure 6 exhibits the competition between the QC effect and the CS effect with diameter decrease of Si QDs and curvature increase on surface doped with Si-O-Si bonds, where the red curve and the black curve respectively describe the QC effect and the CS effect evolution, and the green curve is the last evolution after coupling between the QC effect and the CS effect. The characteristic parameters of the CS effect, such as bonding angle and bonds projection length on surface, are marked along with the black curve in Fig. 6.

**Figure 7:**
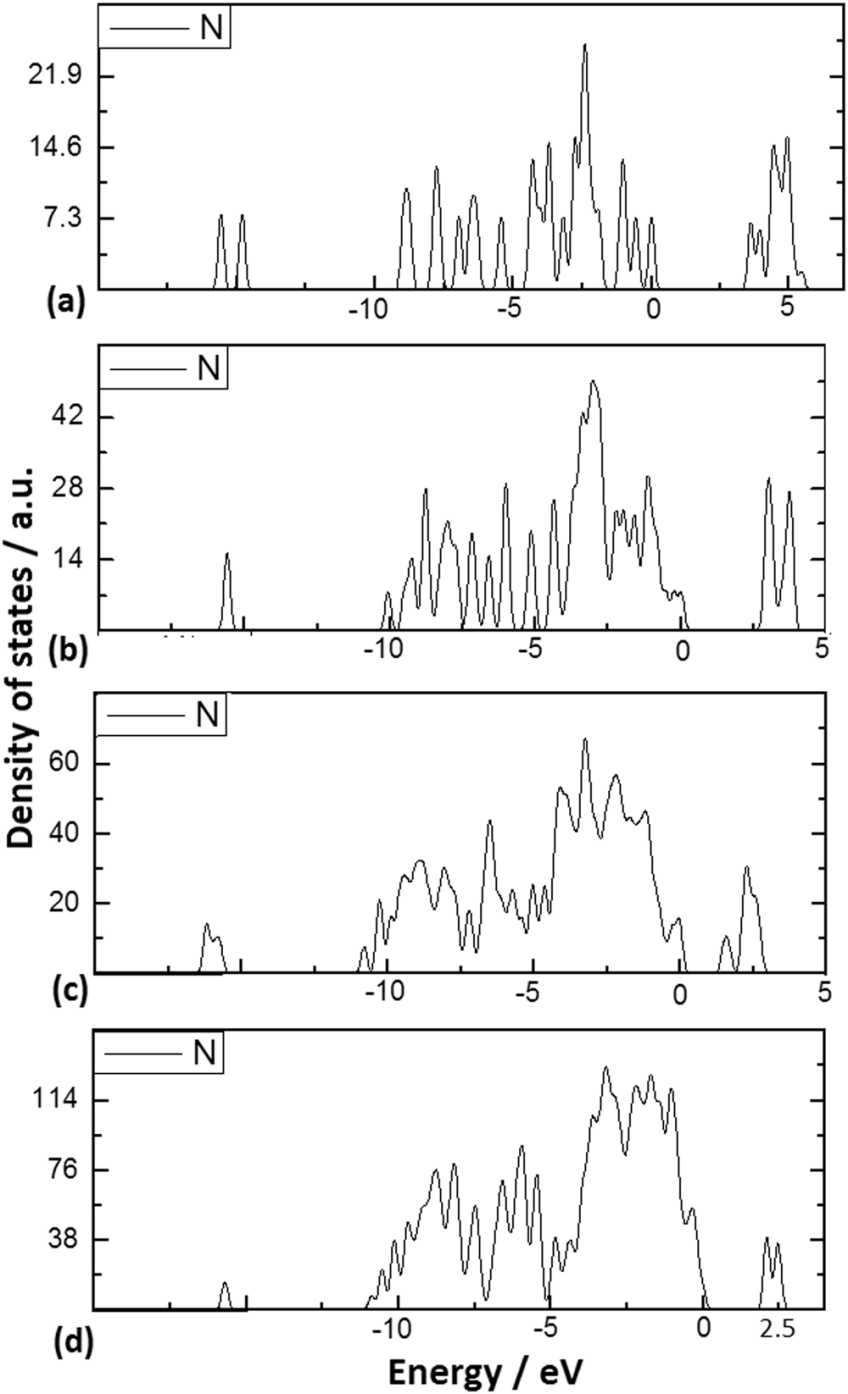
Exhibition of competition between the QC effect and CS effect occurring in Si QDs with Si-N bonds on surface. (**a**) DOS distribution of QDs involving 10 atoms with Si-N bonds on surface. (**b**) DOS distribution of QDs involving 33 atoms with Si-N bonds on surface. (**c**) DOS distribution of QDs involving 66 atoms with Si-N bonds on surface. (**d**) DOS distribution of QDs involving 147atoms with Si-N bonds on surface.

In the results of simulating calculation, the competition between the QC effect and CS effect also occurs in Si QDs bonded with Si-N bonds on surface, as shown in Fig. 7(a,b,c and d) reference to QDs of 10, 33, 66 and 147 Si atoms respectively. Figure 7(a) shows strongest coupling between the QC effect and the CS effect on the QD of 10 Si atoms, where the band gap becomes the widest and the localized states are the most abundant in the band gap.

**Figure 8:**
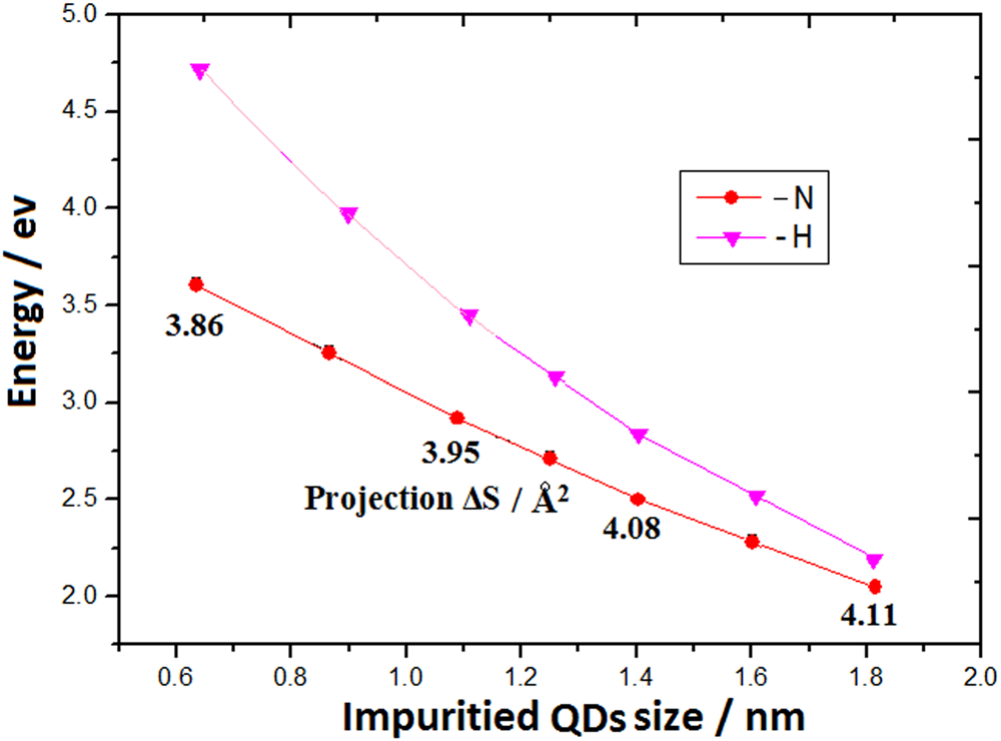
Energy levels evolution curves of Si QDs with Si-H bonds (pink curve) and Si-N bonds (red curve), where the characteristic parameter (bonds projection cover area ΔS) is marked along with it.

The levels evolution after coupling between the QC effect and CS effect is shown along the red curve in Fig. 8, where the characteristic parameter (bonds projection cover area ΔS) is marked along with the red curve, and the pink curve describes evolution in the pure QC effect. Here, in the CS effect formula, parameter **d** equals to 2 and the formula is described as following: E_L_ = C/r^m^ + βB^1/3^ /R, where the bonding cover factor B should be related to bonding angle and projecting area ΔS of Si-N bond on curved surface, in which there is the weaker CS effect owing to the smaller coefficient βB^1/3^.

Making a comparison among the results of the simulating calculation on Si QDs doped with Si = O, Si-O-Si and Si-N bonds on surface, it is discovered that the breaking symmetry of Si = O bond on surface is largest and the breaking symmetry of Si-N bond is smallest on QDs surface in the CS effect. The calculation results are consisted with experimental results, the lower levels of the localized states owing to Si = O bond on QDs surface and their good emission near 700 nm are a good exhibition of the CS effect.

In conclusion, the competition and coupling between the quantum confinement and the breaking symmetry of nanosilicon system are used to manipulate the energy levels and emission wavelength on impuritied nanosilicon, where the CS effect plays a main role. The inverse physical mechanism in the QC effect and the CS effect has been revealed in the study. The results of experiment and calculation demonstrated that the QC effect produces blue-shift of emission wavelength with decrease of Si QDs size, but the CS effect generates emission red-shift with surface curvature increase of impurity atoms bonding on Si QDs, in which the breaking symmetry of nanosystem owing to Si = O, Si-O-Si and Si-N bonding on QDs surface produces the localized states obviously for manipulating the PL emission wavelength. Here, the characteristic parameters of impurity atoms such as bonding angle and projection of bonds on smaller QDs surface have magical affection for manipulating the electronic states in the CS effect. It is interesting to deeply investigate the CS effect coupling with the QC effect in nanosilicon for manipulating emission wavelength, which have a good application in nanolaser on Si chip in future.

## Method

### Simulating calculation

Some physical models have been chosen in order to simulate various kinds of surface structures of nanosilicon doped with impurity atoms. The models based on supercells have advantages that are simple and emphasize the quantum confinement effect and deformation of the surface structure with stoichiometric impurities, in which their electronic behavior is investigated by an ab initio nonrelativistic quantum mechanical analysis. We use the density functional theory (DFT) to calculate the density of states (DOS), which is carried out with the local density approximation (LDA) and gradient-corrected exchange-correlation function (GGA) for the self-consistent total energy methods.
